# Introduction and geographic availability of new antibiotics approved between 1999 and 2014

**DOI:** 10.1371/journal.pone.0205166

**Published:** 2018-10-16

**Authors:** Cecilia Kållberg, Christine Årdal, Hege Salvesen Blix, Eili Klein, Elena M. Martinez, Morten Lindbæk, Kevin Outterson, John-Arne Røttingen, Ramanan Laxminarayan

**Affiliations:** 1 Institute of Health and Society, Faculty of Medicine, University of Oslo, Oslo, Norway; 2 Norwegian Institute of Public Health, Oslo, Norway; 3 School of Pharmacy, University of Oslo, Oslo, Norway; 4 Center for Disease Dynamics, Economics & Policy, Washington, District of Columbia, United States of America; 5 Department of Emergency Medicine, Johns Hopkins School of Medicine, Baltimore, Maryland, United States of America; 6 Department of Epidemiology, Johns Hopkins Bloomberg School of Public Health, Baltimore, Maryland, United States of America; 7 School of Law, Boston University, Boston, Massachusetts, United States of America; 8 CARB-X, Boston, Massachusetts, United States of America; 9 Department of Global Health & Population, Harvard T.H. Chan School of Public Health, Harvard University, Boston, Massachusetts, United States of America; 10 Princeton Environmental Institute, Princeton, New Jersey, United States of America; York University, CANADA

## Abstract

**Background:**

Despite the urgent need for new, effective antibiotics, few antibiotics of value have entered the market during the past decades. Therefore, incentives have been developed to stimulate antibiotic R&D. For these incentives to be effective, geographic availability for recently approved antibiotics needs to be better understood. In this study, we analyze geographic availability and market introduction of antibiotics approved between 1999 and 2014.

**Material and method:**

We identified antibiotics, considered new chemical entities (NCEs) for systemic use approved globally between 1999 and 2014, from national medicine agencies’ lists of approved drugs, and data from the WHO Collaborating Center for Drug Statistics. Geographic availability was mapped using sales data from IQVIA, and analyzed with regards to class, indication, safety, and origin.

**Results:**

Of the 25 identified NCEs, only 12 had registered sales in more than 10 countries. NCEs with the widest geographic availability had registered sales in more than 70 countries within a ten-year timeframe and 30 countries within a three-year timeframe, spreading across five different geographic regions and three country income classes. Half (52%) of the NCEs had an indication for infections caused by antibiotic- resistant bacteria, little diversity was seen regarding target pathogen and indication. Antibiotics originated from and/or marketed by companies from the US or Europe had greater geographic availability compared to Japanese antibiotics, which seldom reached outside of Asia. For 20 NCEs developers chose to fully or partially sublicense marketing rights to a number of companies of different sizes.

**Conclusion:**

Our findings show great variation in geographic availability of antibiotics, indicating that availability in multiple regions and country income classes is possible, but rarely seen within a few years of market authorization. Sublicensing agreements between multiple companies was common practice. Moreover, differences were seen between countries regarding benefit/risk evaluations and company behavior. These findings could be a potential source of uncertainties, and create barriers to assure that working antibiotics are developed and made available according to public health needs.

## Introduction

Extensive use of antimicrobials in humans and animals, in combination with lack of access to clean water, sanitation, and health care has resulted in the accelerated development of antimicrobial resistance [1]. Solving this problem demands a multifaceted solution simultaneously stimulating research and development (R&D) of new treatments, reducing irresponsible use, while assuring access to infectious disease prevention measures and antimicrobials [2].

Over time antibiotic innovation has however slowed considerably [3]. Few new classes have been introduced to market since 2000, none of them targeting Gram-negative bacteria [4]. This indicates that in addition to a decrease in the number of new antibiotics, there is a lack of novel antibiotics targeting some of the most urgent public health needs [5–11]. This is the result of multiple factors. First, antibiotics are considered difficult to develop, both from a scientific and regulatory perspective [12, 13]. Antibiotics are approved using non-inferiority trials, since the use of placebo groups would be neither safe nor ethical. However, non-inferiority trials have a number of weaknesses [14, 15]. While regulatory agencies have addressed many of the challenges such as different regulatory agencies requiring different trial endpoints, demands to conduct multiple clinical trials for different body sites, and different requirements for non-inferiority margins, challenges still remain regarding recruiting patients with clinical infections caused by antibiotic resistance and multidrug resistant bacteria [16]. Second, compared to treatments for chronic diseases, antibiotics are relatively cheap products given for short-term treatments. Moreover, new antibiotics will have to compete with already established products on the market, which can only be done by offering clear patient benefits [17]. Third, the market is unpredictable due to uncertainties about the future prevalence of resistant bacteria, and potential restrictions of antibiotic sales to prohibit unnecessary use [5]. Finally, successful development of an antibiotic may still lead to short-term market presence due to resistance developing only a few years after market entry [18]. Antibiotics are thus considered high-risk projects, with limited market potential, resulting in most big pharmaceutical companies exiting the field and no current system in place to fill the void. This market failure would not be a problem if antibiotics were an inexhaustible resource, but because of antibiotic resistance there is a constant need for new antibiotics. Antibiotic resistance is therefore a scientific challenge as well as the result of a system failure of how we develop and use antibiotics.

To address this problem different interventions have been proposed such as targeted economic incentives, patent pools, extending patents, research networks, private public partnerships, as well as the work to remove barriers in the market approval process [12, 19–21]. This reflects how the process of ensuring availability of effective antibiotics for current and future generations depend on the actions and interaction of multiple stakeholders such as governments, academia, pharmaceutical companies, investors, and regulatory agencies. The geographic pattern and timing of antibiotic availability can be considered to reflect these actions and interactions, in particular decisions related to market authorization and market introduction. However, despite the efforts made so far there is a continued lack of novel antibiotics, indicating that there needs to be more coherence between stakeholders to remove uncertainties and assure that the incentives implemented result in antibiotic of true public health value.

Yet presently limited knowledge exists about global market introduction and geographic availability of antibiotics. While researchers have compared national use of antibiotic classes [22–24], few have focused on individual antibiotics. It has been shown that there are a number of challenges related to market authorization of antibiotics, but often with a focus on one geographic region [25, 26]. In contrast, research addressing multiple regions have focused on drug approval in general [27, 28].

Here, we analyze the introduction and geographic availability of antibiotics, qualifying as new chemical entities for systemic use not previously authorized, to the global market between 1999 and 2014, and discuss factors influencing this process. Our results will contribute to the understanding of potential barriers introducing new antibiotics to the global market.

## Material and methods

To identify antibiotics qualifying as chemical active substances not previously authorized (from here on referred to as new chemical entities or NCEs), we reviewed approval lists of four medicine agencies: the European Medicine Agency’s (EMA) list of registered medicines [29], the United States’ Food and Drug Administration’s (FDA) “Orange book” [30], the Pharmaceuticals and Medical Devices Agency’s (PMDA) list of approved drugs in Japan [31] (lists before 2004 are not available), and the Indian Central Drugs Standard Control Organization (CDSCO) list of approved drugs [32]. EMA and FDA were selected since most new antibiotics are first approved by one of these agencies. PMDA was chosen given that Japan is one of the leading countries in drug development. Finally, we decided to add the CDSCO to assure that antibiotics approved in a large non-high income country was in line with our findings from EMA, FDA and PMDA. NCEs of interest were defined using three inclusion criteria: 1) a first market authorization date between 1999 and 2014, 2) an ATC-code starting with J01 “antibacterials for systemic use”, and 3) receipt of “New Molecular Entity” (NME) status. Because the NME status is only available for products approved by the FDA, we also searched the WHO Collaborating Center for drug statistics methodology (WHOCC) database for NCEs that had applied for an ATC-code [33] in 1999 or later. The WHOCC database is a restricted access database that holds information on all drugs given ATC-codes [34]. This allowed us to identify NCEs not approved in the US. In addition we reviewed published articles reporting on antibiotic R&D, identified by conducting non-systematic searches of publicly available literature [4, 35].

Detailed information about the NCEs; indications including resistant bacteria targets, formulation, and class was obtained from the Summary of Product Characteristics (SPC) provided by the EMA [29], the FDA [36], the Swedish Medical Product Agency (MPA) [37], the UK electronic Medicines Compendium (eMC) [38], Canada’s “Drug Product Database” [39], and the WHOCC ATC-code application database [40]. Using first and last available SPC, from multiple sources, allowed us to produce a more complete list of indications, since approved indications may vary between countries and over time. This resulted in 21 different indications, which were organized into 12 groups (S1 Table).

Safety issues were identified by searching the EMA and FDA webpage sections on post approval safety issues and warnings [29, 36]. NCEs were labeled as having safety issues if there had been 1) toxicity or 2) lack of effect. Toxicity issues included reports of potentially life-threatening adverse effects such as severe liver or severe kidney toxicity, Steven-Johnson syndrome, cardiac arrhythmia etc., leading to withdrawals, label warnings or restrictions of indications. Lack of effect included reports that an NCE had demonstrated lack of effect at the recommended dose, leading to withdrawals, label warnings or restrictions of indications. Warnings concerning packaging and administration issues were not included. Warnings concerning safety issues for specific populations (age groups or individuals with co-morbidity) were not included. In addition we reviewed the “Livertox database”[41] which lists drug induced liver injuries according to a 5 grade severity scale. NCEs that had known cases of liver injury and liver failure (corresponding to level 3–5 on the severity scale) were listed as having safety issues.

Data on year of market authorization, first country of entry (from here on referred to as launch country), company listed as originator and country of origin, company in charge of market introduction, marketing rights agreements, company net sales and number of employees were obtained from the AdisInsight database [42], the Annual Reports in Medicinal Chemistry volume 36–46 [43–56], and other publicly available information such as the official webpages and annual reports of pharmaceutical companies and company press releases (S2 Table). Originator is a term used in the Annual Reports in Medicinal Chemistry for the companies that first patented a chemical entity. Exceptions are made in the case where companies change company name during the patent process or when more than one company is involved in the process. The originator is then decided through an assessment.

To map market entry we acquired sales data from IQVIA (formerly known as QuintilesIMS, before that, IMS Health) [57]. To our knowledge IQVIA currently provides the most extensive source of harmonized data on global antibiotic drug sales. The dataset covers 76 countries in whole or in part from 1999 to 2014. Central America (Costa Rica, El Salvador, Guatemala, Honduras, Nicaragua, and Panama) and French West Africa (Benin, Burkina Faso, Cameroon, Chad, Côte d’Ivoire, Republic of Congo, Guinea, Mali, Niger, Senegal, and Togo) are included as two separate “countries,” as IQVIA reports aggregate sales for these regions. In addition, IQVIA report China, Hong Kong and Taiwan separately. Data from most low-income countries is not available via IQVIA. IQVIA uses national sample surveys of antibiotic sales to develop estimates of the total volume of sales of each NCE (or combination of NCEs) by month or quarter in retail and/or hospital pharmacies in each country. Sixty-six countries had data available for every year between 1999 and 2014, the rest covered partial time periods. Data were provided by IQVIA in both standard units (SUs) and US dollars (USD). SU is an IQVIA designation that represents a single dose unit such as a pill, capsule, or equal amount of liquid. Our analysis utilizes unit-based sales data to indicate if an antibiotic is available in a given country. This should not be confused with regulatory approval, as medicines may be used in countries without regulatory approval via parallel import, likewise countries may have regulatory approval for a medicine but physicians never prescribe it. We therefore use the term “registered sales” instead of “registered” or “market approved” unless there are other supporting sources. The term “geographic availability” denote the number of countries with sales of the specified antibiotic.

We constructed timelines displaying market entry and geographic availability. Patterns of geographic availability were analyzed 3, 5, and 10 years after first market authorization by calculating the distribution of countries (in percent) between different country income classes and geographic regions, as defined by the World Bank [58].

## Results

### Overview of new chemical entities

This study identified 25 NCEs (Table 1) belonging to nine antibiotic classes [40] including two new classes (lipopeptides and oxazolidinones). Thirteen NCEs were broad-spectrum (52%). The most common indications were community-acquired respiratory tract infections (n = 18, 24.7%, including n = 14 for pneumonia), skin and skin structure infections (n = 14, 19%), and urinary tract infections (n = 12, 16%) (S1 Table). Infections caused by resistant bacteria were listed as indications for 13 NCEs (52%), none targeting Gram-negative bacteria. Parenteral formulations were more common than oral formulations (n = 19 compared to n = 13, note that some molecules are available as both parenteral and oral formulation). The majority of NCEs were originated by Japanese (n = 11) or US companies (n = 6). The majority of NCEs were launched in Japan (n = 7) or the US (n = 12). Only 15 NCEs were launched in the country where the originator is based. FDA reported post-approval safety issues for eight NCEs (daptomycin, doripenem, gatifloxacin, gemifloxacin, linezolid, moxifloxacin, telithromycin, tigecycline) leading to market withdrawal for one NCE (gatifloxacin) and restrictions on indications for two NCEs (doripenem and telithromycin). EMA reported safety issues for eight NCEs (ceftobiprole, doripenem, garenoxacin, gemifloxacin, moxifloxacin, telavancin, telithromycin, tigecycline). The EMA rejected market authorization for ceftobiprole, garenoxacin and gemifloxacin, and restricted indications for one NCE (tigecycline). The EMA has not evaluated gatifloxacin and linezolid. (Note that ceftobiprole was later approved by individual European countries while ceftobiprole and garenoxacin are not approved by the FDA, we do not know if FDA has received applications for these two). Two NCEs were withdrawn from one or more markets by the pharmaceutical companies holding marketing rights (doripenem and telithromycin). For more information see S3 Table. Five NCEs (ceftaroline, gemifloxacin, linezolid, moxifloxacin, telithromycin) had severe liver toxicity reported as a safety issue. One NCE (telavancin) had severe kidney toxicity reported as a safety issue. Fluoroquinolones reported safety issues on a class level.

**Table 1:** Antibiotics approved between 1999 and 2014.

ATC-code	NCE	MA year^a^	Year of first sale	Form^b^	Class	Number of indications	Spectrum and resistance	Safety issues reported by the EMA or the FDA	Country of originator	Originator	Launch country	Developer	Countries with registered sales
J01FG02	dalfopristin/ quinupristin	1999	2005	O, P	streptogramins	4	G+, VREF (bacteremia, removed in 2010)	not reported	France	Rhone-Poulenc-Rorer	UK	Aventis	**21**
J01MA16	gatifloxacin	1999	1999	O, P	fluoroquinolones	3	G+, G-	FDA: withdrawal due to severe cases on hypo-and hyperglycemia (2006)	Japan	Kyorin Pharmaceuticals	US	Bristol-Myers Squibb	**30**
J01MA14	moxifloxacin	1999	1999	O, P	fluoroquinolones	5	G+, G-, MDRSP	EMA: rerefferal due to liver toxicity, side effects in nervous system and muscles, prolonged QT-interval (2008); FDA: permanent side effects of the tendons, muscles, joints, nerves, and central nervous system (2016) Livertox: liver failure is rare but has occurred	Germany	Bayer	Germany	Bayer	**75**
J01XX08	linezolid	2000	2000	O, P	oxazolidinones^c^	3	G+, MRSA (nosocomial pneumonia, SSTI), VREF (bacteremia)	FDA: myelosuppression (2001); Livertox: rare instances of lactic acidosis and liver injury	US	Pharmacia Corp.	US	Pharmacia Corp.	**70**
J01FA15	telithromycin	2001	2001	O	macrolides	1	G+, G-, MDRSP	EMA: liver toxicity, QT-prolongation (2007); FDA: liver toxicity, loss of consciousness and visual disturbances; Livertox: several cases of severe drug induced liver injury (2006)	France	Aventis	Germany	Aventis	**43**
none	balofloxacin	2002	2006	O	fluoroquinolone	2	G+	missing	Japan	Chugai Pharmaceutical	South Korea	Choongwae (now JW pharmaceuticals)	**3**
J01DH05	biapenem	2002	2002	P	carbapenem	4	G+, G-	missing	Japan	Wyeth KK	Japan	Meiji Seika	**3**
J01DH03	ertapenem	2002	2002	P	carbapenems	5	G+, G-	not reported	UK	Astra Zeneca	US	Merck	**65**
J01MA18	pazufloxacin	2002	2002	P	fluoroquinolones	5	G+, G-	missing	Japan	Toyama Chemical	Japan	Toyama Chemical/ Mitsubishi	**3**
J01MA17	prulifloxacin	2002	2002	O	fluoroquinolone	6	G+, G-	see moxifloxacin	Japan	Nippon Shinyaku	Japan	Nippon Shinyaku and Meiji Seika	**14**
J01XX09	daptomycin	2003	2003	P	lipopeptides^c^	3	G+, MRSA (SSTI, bacteremia)	FDA: eosinophilic pneumonia (2010)	US	Eli Lilly	US	Cubist	**47**
J01MA15	gemifloxacin	2003	2003	O, P	fluoroquinolones	2	G+, G-, MDRSP	EMA: application rejected due to genotoxicity (2009); FDA: see moxifloxacin Livertox: rare instances of acute liver injury	South Korea	LG Life Sciences	US	Oscient	**28**
J01DH04	doripenem	2005	2005	P	carbapenems	3	G-	EMA: non-sufficient dosing for VAP (2012); FDA: increased death and lower clinical cure rate in VAP compared to alternative (2014)	Japan	Shionogi	Japan	Shinogi/ Peninsula (owned by J&J)	**44**
J01AA12	tigecycline	2005	2005	P	tetracycline	3	G+, G-, MRSA (SSTI and IAI)	EMA: effectiveness in severe CAP (2008); FDA: increased risk of death with iv (2010)	US	Wyeth	US	Wyeth	**65**
J01MA19	garenoxacin	2007	2007	O, P	fluoroquinolones	4	G+, G-	EMA: not approved due to hypotension, effect in blood glucose (2007)	Japan	Toyama Chemical	Japan	Toyama /Taisho/Astellas	**2**
J01DI01	ceftobiprole	2008	2008	P	cephalosporin	3	G+, MRSA (CAP)	EMA: not approved due to non-compliance with "good clinical practice" (2010) (note late approved in individual countries)	Switzerland	Roche	Canada	Basilea/ J&J	**5**
J01MA21	sitafloxacin	2008	2008	O	fluoroquinolones	4	G+, G-	missing	Japan	Daiichi Sankyo	Japan	DaiichiSankyo	**2**
none	tebipenem	2009	2009	O	carbapenems	1	G+, G-	missing	Japan	Wyeth KK	Japan	Meiji Seika Pharma	**1**
J01XA03	telavancin	2009	2009	P	glycopeptides	2	G+, MRSA (SSTI, HAP/VAP)	EMA: kidney problems and QT-prolongation (2008)	US	Theravance	US	Theravance/ Astellas	**1**
none	antofloxacin	2010	2010	O	fluoroquinolone	3	G+, G-	missing	China	Shanghai Institute Of Materia Medica	China	Anhui global pharmaceutical	**1**
J01DI02	ceftaroline	2010	2011	P	cephalosporin	2	G+, MRSA (SSTI)	Livertox: rare cases of apparent liver injury	Japan	Takeda	US	Forest Laboratories Inc.	**31**
J01DI54	ceftolozane/ tazobactam	2014	2014	P	cephalosporin	2	G-	not reported	Japan	Astellas	US	Cubist	**1**
J01XA04	dalbavancin	2014	2014	P	glycopeptides	1	G+, MRSA (SSTI)	not reported	US	Vicuron Pharmaceuticals	US	Durata (spin off from Pfizer)	**1**
J01XA05	oritavancin	2014	2014	P	glycopeptides	1	G+, MRSA (SSTI)	not reported	US	Eli Lilly	US	The Medicines Company	**1**
J01XX11	tedizolid	2014	2014	O, P	oxazolidinone	1	G+, MRSA (SSTI)	not reported	South Korea	Dong-A Pharmaceutical	US	Cubist	**2**

Antibiotic molecules considered new chemical entities approved between 1999 and 2014. CAP = Community-Acquired Pneumonia, HAP = Hospital-Acquired Pneumonia, IAI = Intra-Abdominal Infections, MDRSP = Multi-Drug Resistant Streptococcus Pneumoniae, MRSA = Methicillin-Resistant Staphylococcus Aureus, SSTI = Skin and Soft Tissue Infections, VAP = Ventilator-Associated Pneumonia, VREF = Vancomycin-Resistant Enterococcus Faecium

a Year of market authorization

b Form stands for formulation and is coded O for oral and P for injectable

c New antibiotic class

### Geographic availability

**Fig 1:**
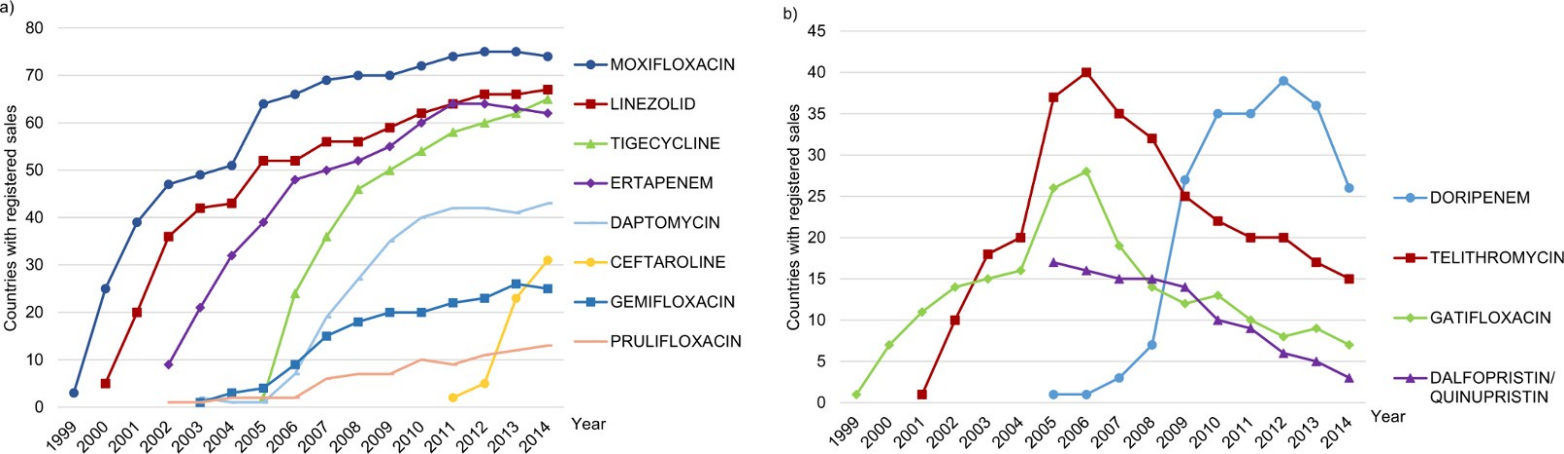
Number of countries with registered sales of the NCEs reaching more than 10 countries. (a) The 8 NCEs experiencing a continuous increase in number of countries with registered sales per year. (b) The 4 NCEs experiencing an increase followed by a decrease in number of countries with registered sales per year.

The 25 NCEs had registered sales from one to 75 countries (Table 1). Of these, 21 NCEs were approved no later than 2010 (resulting in a minimum market presence of four years) allowing analysis of geographic availability over time. (The remaining four NCEs were approved in 2014, and only available in one country). Twelve of the 21 NCEs (57%) had registered sales in over 10 countries (Fig 1A and 1B), while nine NCEs had limited geographic availability with registered sales in five countries or less.

The originating companies for the 12 NCEs with greater geographic availability were split quite evenly between Japan (n = 4, 33%), Europe (n = 4, 33%), and the US (n = 3, 25%). More than half of the NCEs (n = 7, 58%) were launched in the US. Originating and launch country differed for six NCEs (50%). Ertapenem, linezolid, moxifloxacin, and tigecycline had the greatest geographic availability, reaching more than 60 countries by 2014, followed by daptomycin, reaching 43 countries by 2014. All five NCEs were originated by European or US companies and launched in Europe or the US. Having an indication for infections caused by antibiotic resistant bacteria was common for NCEs with greater geographic availability (eight of the top 12 NCEs, 67%, and three of the top four NCEs, 75%).

Of the 9 NCEs available in five countries or less (excluding 4 out of the 13 NCEs reaching five countries or less, because they were registered in 2014), seven (78%) originated by Asian companies (six by Japanese companies), and seven (78%) had been launched in Asia (five in Japan). Origin and launch country differed for two NCEs (22%) (Table 1). Only two (22%) had indications for antibiotic resistant bacteria.

NCEs with greater geographic availability had 1–6 indications (mean 3.3, median 3), similar to NCEs with limited geographic availability which had 1–5 indications (mean 3.1, median 3). Safety issues were reported more often for NCEs with greater geographic availability (10 out of 12 NCEs) compared to NCEs with limited geographic availability (3 out of 9 NCEs). Spectrum, formulation (Table 1) and type of indication (S1 Table) did not seem to be drivers of differences in geographic availability between the two groups. NCEs that were introduced earlier had greater geographic availability.

### Company size and management of marketing rights

We examined the size of companies responsible for development and marketing of the NCEs, according to the number of employees and net sales in the year of market introduction (S4 Table). We found that the majority (88%) of these companies had a turnover of more than 50 million euros and more than 250 employees, which is the cut-off line for SMEs according to the current EU definition, [59]. Hence, SMEs did not have a major role in market introduction of the NCEs. However, we found that smaller companies were involved in marketing the NCEs in additional regions/countries after first market introduction had taken place (S2 Table).

Sublicensing marketing agreements between companies seemed to be a common practice, with three main trends: originators sublicensing full marketing rights to other companies (n = 11), originators choosing to keep global marketing rights (n = 5), and originators combining the two approaches, i.e. keeping marketing rights in specific areas (most often the domestic market) while sublicensing remaining areas to other companies (n = 9) (S2 Table). Changes in ownership of marketing rights also took place in the form of mergers and acquisitions between companies. Amongst the 12 NCEs with greater geographic availability (Fig 1A and 1B), three of top four NCEs (linezolid, moxifloxacin, tigecycline) were originated and marketed by the same pharmaceutical companies (with the exception of the marketing of moxifloxacin in Japan) as well as the seventh highest ranking for availability (telithromycin). The top four NCEs have been on the market since 2005. The remaining nine NCEs (available in more than 10 countries) were fully or partly sublicensed to either multiple companies (n = 5), or to one other pharmaceutical company (n = 4). These companies were in most cases based in Europe or the US. Japanese NCEs sublicensed to US companies had a greater geographic availability compared to NCEs marketed by Japanese companies. All but two NCEs with limited geographic availability (excluding NCEs marketed in 2014) were marketed by Asian companies, with none marketed outside of Asia.

### Speed of introduction

The four NCEs with greatest geographic availability (moxifloxacin, linezolid, ertapenem, tigecycline) had registered sales in over 30 countries within three years after first market authorization. This level of geographic availability was greater than any other NCE in any three-year time period. The only other NCE with a similar pattern was ceftaroline, approved in 2011, somewhat later compared to the top four. Other drugs, such as daptomycin and doripenem, were rolled out more slowly, with sales being registered only in the launch country for about three years before sales were registered in other countries. Dalfopristin/quinupristin, doripenem, gatifloxacin, and telithromycin were the only NCEs showing a gradual decrease in availability, three of which had their market authorization withdrawn due to safety issues. Eight other NCEs experienced safety issues without having a similar impact on geographic spread to other countries.

### Pattern of reach

Table 2 and Table 3 show the introduction of NCEs based on geographic region and country income classes 3, 5, and 10 years after first market authorization, for NCEs with registered sales in more than 10 countries. Ertapenem, linezolid, moxifloxacin, and tigecycline were the only NCEs that had market presence after three years in 5–6 geographic regions and across all country income classes registered. Apart from these four, market introduction for most NCEs initially took place in high-income countries, either the US or countries in Europe, gradually spreading through South America and East Asia and the Pacific. Introduction of NCEs in the Middle East and North Africa (MENA), South Asia, and Sub-Saharan African Regions largely occurred after the spread to higher income countries. The majority of countries (90%) had registered sales of 2 to 10 NCEs. The US had registered sales of the highest number of NCEs (n = 16), followed by Japan (n = 14), India, China, and Puerto Rico (n = 12). Europe and the MENA showed relatively homogenous registered sales across countries.

**Table 2:** Spread of NCEs between country income classes.

NCE	Year of MA	Year after first MA	High Income Countries	Upper Middle Income Countries	Lower Middle Income Countries	Countries with registered sales	Total number of countries with registered sales	Span	Code
MOXIFLOXACIN	1999	3	67%	26%	5%	39	75	0–10	
		5	65%	29%	4%	49		11–20	
		10	59%	27%	13%	70		21–30	
LINEZOLID	2000	3	81%	17%	3%	36	70	31–40	
		5	72%	23%	5%	43		41–50	
		10	58%	27%	14%	59		51–60	
ERTAPENEM	2002	3	63%	31%	3%	32	65	61–70	
		5	65%	29%	4%	48		71–80	
		10	53%	33%	13%	64		81–90	
TIGECYCLINE	2005	3	69%	25%	6%	36	65	91–100	
		5	64%	26%	8%	50			
		10	55%	29%	14%	65			
DAPTOMYCIN	2003	3	100%	0%	0%	1	47		
		5	95%	5%	0%	19			
		10	62%	29%	10%	42			
DORIPENEM	2005	3	100%	0%	0%	3	44		
		5	70%	22%	7%	27			
		10	50%	27%	19%	26			
TELITHROMYCIN	2001	3	72%	28%	0%	18	43		
		5	61%	34%	3%	38			
		10	82%	14%	0%	22			
CEFTAROLINE	2010	3	78%	17%	4%	23	31		
GATIFLOXACIN	1999	3	45%	45%	9%	11	30		
		5	47%	40%	13%	15			
		10	21%	29%	43%	14			
GEMIFLOXACIN	2003	3	75%	25%	0%	4	28		
		5	40%	40%	20%	15			
		10	35%	30%	35%	23			
DALFOPRISTIN/ QUINUPRISTIN	1999	3	100%	0%	0%	15	21		
		5	100%	0%	0%	14			
		10	100%	0%	0%	3			
PRULIFLOXACIN	2002	3	100%	0%	0%	2	14		
		5	100%	0%	0%	2			
		10	67%	22%	11%	9			

Spread of NCEs distributed between country income classes 3, 5, and 10 years after first market authorization (MA). NCEs reaching less than 10 countries are not included.

**Table 3:** Spread of NCEs between geographic regions.

NCE	Year of MA	Year after first MA	East Asia & Pacific	Europe & Central Asia	MENA	North America	Latin America & Caribbean	South Asia	Sub-Saharan Africa	Countries with registered sales	Total number of countries with registered sales
MOXIFLOXACIN	1999	3	21%	41%	8%	5%	23%	0%	3%	39	75
		5	20%	43%	8%	4%	22%	0%	2%	49	
		10	19%	44%	10%	3%	17%	4%	3%	70	
LINEZOLID	2000	3	22%	50%	0%	6%	17%	3%	3%	36	70
		5	23%	51%	0%	5%	16%	2%	2%	43	
		10	22%	46%	2%	3%	19%	7%	2%	59	
ERTAPENEM	2002	3	25%	38%	0%	6%	28%	0%	3%	32	65
		5	23%	46%	2%	4%	23%	0%	2%	48	
		10	19%	48%	6%	3%	19%	3%	2%	64	
TIGECYCLINE	2005	3	19%	44%	6%	6%	22%	3%	0%	36	65
		5	20%	46%	4%	4%	20%	4%	2%	50	
		10	20%	46%	8%	3%	17%	5%	2%	65	
DAPTOMYCIN	2003	3	0%	0%	0%	100%	0%	0%	0%	1	47
		5	0%	84%	0%	5%	11%	0%	0%	19	
		10	26%	52%	0%	5%	14%	2%	0%	42	
DORIPENEM	2005	3	33%	0%	0%	33%	33%	0%	0%	3	44
		5	26%	59%	0%	4%	7%	4%	0%	27	
		10	35%	38%	0%	4%	15%	4%	4%	26	
TELITHROMYCIN	2001	3	6%	61%	17%	0%	11%	0%	6%	18	43
		5	11%	39%	13%	5%	29%	0%	3%	38	
		10	5%	68%	0%	9%	9%	0%	9%	22	
CEFTAROLINE	2010	3	17%	74%	0%	4%	4%	0%	0%	23	31
GATIFLOXACIN	1999	3	36%	0%	0%	18%	36%	0%	9%	11	30
		5	47%	0%	0%	13%	27%	7%	7%	15	
		10	36%	7%	7%	7%	21%	21%	0%	14	
GEMIFLOXACIN	2003	3	25%	0%	0%	25%	25%	0%	25%	4	28
		5	20%	7%	20%	13%	20%	13%	7%	15	
		10	13%	13%	35%	4%	17%	13%	4%	23	
DALFOPRISTIN/	1999	3	20%	60%	0%	13%	7%	0%	0%	15	21
QUINUPRISTIN											
		5	21%	64%	0%	7%	7%	0%	0%	14	
		10	33%	33%	0%	33%	0%	0%	0%	3	
PRULIFLOXACIN	2002	3	50%	50%	0%	0%	0%	0%	0%	2	14
		5	50%	50%	0%	0%	0%	0%	0%	2	
		10	33%	56%	0%	0%	0%	11%	0%	9	

Spread of NCEs distributed between geographic regions 3, 5, and 10 years after first market authorization (MA). NCEs reaching less than 10 countries are not included.

### Fluoroquinolones

Nine of the 25 NCEs belonged to the fluoroquinolone class. Three NCEs in this class were available in 28 countries or more: moxifloxacin (made available first of the fluoroquinolones in the dataset, 75 countries), followed by gatifloxacin (made available as the second of the fluoroquinolones, 30 countries) and gemifloxacin (made available as the sixth of the fluoroquinolones, 28 countries). These NCEs were available as both parenteral and oral formulations. Five of the remaining 6 NCEs were available in three countries or less (the exception being prulifloxacin, n = 14). Four of these were oral formulations, one parenteral and one both parenteral and oral. Indications were similar for all nine fluoroquinolones. However, only two of the nine had indications for multi-drug resistant bacteria. Gemifloxacin was labeled for penicillin-resistant strains in 2003 and multi-drug resistant streptococcus pneumoniae (MDRSP) in 2004, while moxifloxacin was labeled for penicillin-resistant strains in 2003 and MDRSP in 2005. Moxifloxacin was originated and launched in Germany. Gemifloxacin originated from South Korea and gatifloxacin from Japan, both were launched in the US. The remaining NCEs originated from and were launched in Asia (foremost Japan).

## Discussion

In this study we analyzed the introduction and geographic availability of antibiotics approved between 1999 and 2014. We identified that antibiotics, for systemic use, can reach as many as 70 national markets within a ten-year timeframe and 30 countries within a three-year timeframe, spreading across different geographic regions including both high- and middle-income settings. However, geographic availability was remarkably variable, with only 12 NCEs having registered sales in more than 10 countries. This indicates that availability in multiple regions and country income classes is possible, but rarely seen within a few years of market authorization.

We use the term “geographic availability” to denote the number of countries with sales of the specified NCE, which can not be used to determine if the NCE is appropriately available to patients with a clinical need. Overuse of antibiotics is often addressed as one of the main reasons for development of antibiotic resistance. Lack of availability, and therefore access, is however equally problematic since this has been shown to lead to increased use of inappropriate treatments [60]. Moreover, more people still die from lack of access to antibiotics than from antibiotic resistance [61]. It is therefore possible that some of the NCEs researched in this paper should be made available in more countries, while the availability of some NCEs should be more restricted. Likewise, we do not know of the true clinical value of the antibiotics researched. Widespread geographic availability could be the result of the fact that the antibiotic fill a medical need, or the result of a successful marketing strategy. However, limited geographic availability would probably not occur if the antibiotic had a current clinical value.

Little diversity regarding target pathogen and indication was seen amongst the 25 NCEs we identified. We found that over half (52%) of the NCEs had an indication for infections caused by antibiotic resistant bacteria, potentially indicating that the R&D pipeline has, to some extent, been responsive to the clinical need for antibiotics targeting antibiotic resistant bacteria. Unfortunately, these NCEs cover the same three Gram-positive pathogens (MRSA, VREF, MDRSP), leaving Gram-negative resistant bacteria unattended. As estimated time from patent to clinical approval of a drug is approximately 10 years, the number of drugs focused on Gram-positive bacteria, particularly MRSA, is understandable as these were first labeled “superbugs” 10–20 years ago. Furthermore, target indication for these drugs was dominated by community-acquired respiratory tract infections, skin and skin structure infections, and urinary tract infections which began rapidly increasing in the early 2000s [62]. This suggests that incentives to stimulate antibiotic R&D need to consider how to create diversity regarding target indications and pathogens. This should be considered in addition to the main objective; to assure that incentives to stimulate R&D of antibiotics offer added patient benefits for current and future patients, targeting infectious diseases of great public health concern.

It is reasonable to assume that safety issues could have an impact on geographic availability. In contrast to what might be expected, we found that safety issues were more common amongst NCEs with greater geographic availability. We suspect this is due to not only increased number of users, but also difficulties accessing safety documentation for NCEs outside of the US or Europe, combined with the greater visibility of safety issues of NCEs consumed in multiple countries. We also observed differences in EMA and FDA evaluations. For example, in the case of doripenem, EMA decided to issue a warning only, while FDA removed ventilator-associated pneumonia (VAP) as an indication. The drug was later withdrawn from the European market. In the case of telithromycin, both EMA and FDA raised concerns about safety issues which lead the FDA to remove the indication for acute exacerbation of chronic bronchitis and sinusitis, and later a withdrawal from the US market by the company holding marketing rights. EMA on the other hand, kept the same indications but recommended that telithromycin only be used when there is a high risk for resistant bacteria. Another example is tigecycline. Here, FDA issued a black box warning due to concerns about increased risk of death when tigecycline is administered intravenously. In comparison, EMA instead raised concerns regarding the efficacy of tigecycline when given for community-acquired pneumonia (CAP) leading to removal of this indication within Europe. Finally, both EMA and FDA approved telavancin for hospital-acquired pneumonia (HAP)/VAP caused by MRSA, while EMA did not include skin infections as an indication. These examples demonstrate how weighing patient benefit against safety issues will differ depending on the county/regional setting [63], which could create unpredictability for companies developing antibiotics.

We observed differences between regions concerning company behavior. According to our analysis, antibiotics originated from and/or marketed by companies from the US or Europe had greater geographic availability compared to Japanese antibiotics. Historically, Japan has been one of the major antibiotic developers in the world [64], and accounted for a third of the NCEs in our dataset. However, the Japanese NCEs made up the majority of NCEs reaching less than 5 countries. This is in line with research by Tsuji et al. showing that Japanese NCEs are made available in fewer markets than those from the US or Europe [27]. We do not know if this is the result of failed attempts by Japanese companies to obtain market authorization outside of Asia, or if no attempts have been made. However, we do know that only one of the Japanese NCEs has been evaluated through the EMA centralized authorization procedure (S3 Table). One possible explanation could be differences in regulatory requirements, including requirements for clinical trials conducted on specific populations. Research shows that there are clear differences between medicine agencies in Europe, the US and Japan with respect to approval times and number of NCEs approved [65], which might discourage Japanese companies from seeking market authorization outside of Asia. It is therefore positive that the major regulatory agencies have increased their collaboration over the years to address some of these issues. Alternatively, the lack of spread of Japanese antibiotics could be the result of marketing strategies, with companies focusing on their domestic market. It should be noted that the majority of NCEs with limited reach were fluoroquinolones. Ultimately, investments in and experience from Japanese antibiotic R&D did not reach the global market. While there is little interest in “me too” fluoroquinolones, it is in the public interest to assure that researchers with experience and knowledge of antibiotic R&D receives enough support to assure that they are able to continue working in this field.

Finally, we observed that developers most often chose to fully or partially sublicense marketing rights to a number of companies of different sizes. Therefore, launching NCEs in different geographic regions and country income classes often depended on multiple companies. In many cases even the big markets (Europe, Japan and the US) were controlled by different companies. This observation is particularly relevant for the debate on the use of economic incentives (which is one of the incentives currently suggested to stimulate R&D of new antibiotics), and raises the question whether economic incentives would have to be divided between multiple companies if they are to stimulate responsible use and access. If this is the case it will be important to consider what the size of these incentives should be since dividing it creates the risk of reducing the payout to individual stakeholders so much as to make the incentive unattractive for drug developers and thereby ineffective. In addition, dealing with multiple companies might make it difficult to know who is operating where in collaboration with whom, and whom to hold responsible. Furthermore, since sublicensing seems to be a common way of operating, it might in fact be the value of these sublicensing agreements that new economic incentives will have to compete against, if they are to be considered an option by the industry. Of particular concern is also how to ensure that an obligation to ensure geographic availability does not deter the interest from small- and medium-sized companies—who may not have the capacity to market an antibiotic in multiple geographic areas—to engage in antibiotic R&D. This would be regrettable given that the path from the bench to bedside often requires many changes in ownership, and an analysis of the most recent NCE approvals found that SMEs played key roles in pre-launch R&D [3]. With the current exit of big pharmaceutical companies there is a need to discuss how to fill their place. Interventions developed to incentivize the industry to reinvest in antibiotic R&D is an option, but could prove to not be able to compete with the benefits companies expect when developing other types of drugs. Moreover, the current system for antibiotic R&D does not sufficiently address responsible use and access. Therefore, there is a need to also consider alternatives for antibiotic R&D. Whether these alternatives should include SME’s, academia or the public sector is beyond the scope of this article.

Four main limitations should be considered when interpreting our findings. First, depending on national data collection systems the IQVIA data coverage differs between countries and sectors, potentially resulting in inaccuracies in the data set. As mentioned previously, IQVIA does not collect data from many low-income countries. Second, when acquiring the data, we chose to include registrations under the ATC-code J01 only. To our knowledge, this excluded four additional antibiotics: besifloxacin (S01AE08 ophthalmology), fidaxomicin (A07AA12 intestinal infections), retapamulin (D06AX13 dermatology), and rifaximin (A07AA11 intestinal infections and D06AX11 dermatology). Third, our approach to identifying NCEs focused on the activities of the medicine agencies in Europe, Japan, India, and the US. There is therefore a risk that we have overlooked NCEs from other countries that should have been included. We partially addressed this issue by including various reports on drug approval in our search [4, 35]. Fourth, we were unable to include data from non-English data sources, making it difficult to obtain detailed information about antibiotics not approved in Europe or the US. Finally, we would like to acknowledge the importance of research and development of alternative treatments and rapid diagnostic tests that offers additional mechanisms to combat AMR. This is however outside of the scope of this article.

## Conclusion

Our findings demonstrate that antibiotics, for systemic use, can reach as many as 70 national markets within a ten-year timeframe and 30 countries within a three-year timeframe, spreading across different geographic regions including both high- and middle-income settings. Yet the geographic availability is highly variable. More than half of the NCEs were approved for infections caused by antibiotic resistant bacteria, however little diversity was seen regarding target pathogen and indication, demonstrating the need to incentivize diversity in antibiotic R&D. Sublicensing agreements between different sized companies were common, making geographic availability a shared responsibility between multiple-companies. Moreover, differences were observed between countries regarding benefit/risk evaluations and company behavior. The impact of these differences on geographic availability is difficult to assess, but could be a potential source of uncertainties for companies involved in antibiotic R&D, and create barriers to assure that working antibiotics are developed and made available according to public health needs. Additional research is needed to explore geographic availability from the perspective of added health benefit and clinical value of new antibiotics, including further assessment of factors that determine stakeholder decision-making regarding geographic availability based on public health needs.

## Supporting information

S1 Table Indications for the 25 NCEs grouped in 12 main indication groups. (PDF)

S2 Table Companies in charge of development and marketing. (PDF)

S3 Table EMA and FDA market authorization dates. (PDF)

S4 Table Company size measured in net sales and number of employees. (PDF)
