# Supplementary material for: Introduction and geographic availability of new antibiotics approved between 1999 and 2014
Source: PLoS One. 2018 Oct 16;13(10):e0205166. doi: 10.1371/journal.pone.0205166 (PMC6191083; doi:10.1371/journal.pone.0205166)
Supplement: S1 Table — (PDF) [file pone.0205166.s001.pdf]

S1 Table. Indications for the 25 NCEs grouped in 12 main indication groups

ATC-code	NCE	Community acquired respiratory tract infections[1]	Nosocomial respiratory tract infections[2]	Skin and soft tissue infections[3]	Urinary tract infections[4]	Complicated intra-abdominal infections	Bloodstream infections (bacteremia)	Right-side infective endocarditis	Secondary infection after trauma, burn or surgical wounds	Osteitis	Plague	Reproductive tract infections[5]	Gonorrhea
none	antofloxacin	1			1				1				
none	balofloxacin	1			1								
J01DH05	biapenem[6]	1			1	1	1						
J01DI02	ceftaroline	1		1									
J01DI01	ceftobiprole[7]	1	1	1									
J01DI54	ceftolozane				1	1							
J01XA04	dalbavancin			1									
J01XX09	daptomycin[8]			1			1	1					
J01DH04	doripenem[9]		1		1	1							
J01DH03	ertapenem[10]	1		1	1	1						1	
J01MA19	garenoxacin	1		1		1						1	
J01MA16	gatifloxacin	1			1								1
J01MA15	gemifloxacin[11]	1			1								
J01XX08	linezolid[12]	1	1	1									
J01MA14	moxifloxacin[13]	1		1		1					1	1	
J01XA05	oritavancin			1									
J01MA18	pazufloxacin	1			1	1			1			1	
J01MA17	prulifloxacin	1		1	1	1			1			1	
J01FG02	quinupristin/ dalfopristin[14]	1		1	1	1							
J01MA21	sitafloxacin	1			1					1		1	
none	tebipenem	1											
J01XX11	tedizolid			1									
J01XA03	telavancin[15]		1	1									
J01FA15	telithromycin[16]	1											
J01AA12	tigecycline[17]	1		1		1							
**Total:**		**18**	**4**	**14**	**12**	**10**	**2**	**1**	**3**	**1**	**1**	**6**	**1**

1. Community acquired respiratory tract infections include community acquired pneumonia, acute exacerbation of chronic bronchitis, acute maxillary sinusitis, pharyngitis/tonsillitis
2. Nosocomial respiratory tract infections include nosocomial pneumonia, hospital-acquired pneumonia (HAP)/ ventilator-acquired pneumonia (VAP)

3. Skin infections include acute bacterial skin and soft tissue infections, uncomplicated skin and soft tissue infections, complicated skin and soft tissue infections
4. Urinary tract infections include uncomplicated urinary tract infections, complicated urinary tract infections, pyelonephritis
5. Reproductive tract infections include mild to moderate pelvic inflammatory disease, acute pelvic infections including postpartum endomyometritis, septic abortion and post-surgical gynecologic infections
6. Indications include the indications listed on ATC-application to the WHOCC
7. EMA rejected skin and soft tissue infections as an indication in 2010, indication was approved by Sweden in 2013 and Canada in 2015
8. FDA added blood stream infections in 2006
9. EMA approved VAP as an indication in 2008, sent out warning in 2012 that dosage might not be enough to treat VAP. FDA removed VAP as an indication
10. Approved for urinary tract infections by the FDA
11. FDA changed the indication for penicillin- resistant infections to multi- drug resistant infections in 2004
12. Mutual recognition approval in Europe. Sweden and UK did not add uncomplicated skin infections as an indication
13. FDA added plague as an indication based on in vitro studies. EMA have not approved intra-abdominal infections as an indication. FDA changed the indication for penicillin- resistant infections to multi- drug resistant infections in 2005
14. FDA removed VREF as an indication in 2010, Sweden and Canada took it off market in 2008
15. EMA have not approved skin infections as an indication due to concerns for kidney toxicity but approved for MRSA in nosocomial pneumonia due to lack of alternative treatments
16. FDA removed indications for chronic bronchitis or sinusitis in 2007 due to liver toxicity but indications for community acquired pneumonia was kept. The company took the product of the market shortly after. EMA decided to keep these indications only to be used" in the event of resistance also indicated for tonsillitis, sinusitis, exacerbation for chronic exacerbation.
17. Community acquired pneumonia and intra-abdominal infections were added by the FDA in 2009. EMA and Canada have not approved community acquired pneumonia as an indication
