# Supplementary material for: Introduction and geographic availability of new antibiotics approved between 1999 and 2014
Source: PLoS One. 2018 Oct 16;13(10):e0205166. doi: 10.1371/journal.pone.0205166 (PMC6191083; doi:10.1371/journal.pone.0205166)
Supplement: S2 Table — (PDF) [file pone.0205166.s002.pdf]

S2 Table. Companies in charge of development and marketing

NCE	MA year	Countries reached	Companies in charge of development and marketing
Antofloxacin	2010	1	originated by Shanghai Institute of Materia Medica (SIMM), Chinese Academy of Sciences (CAS) and Anhui Global Pharmaceutical, Inc. (http://english.simm.cas.cn/rp/200906/t20090626_9128.html) (HQ China), marketed in China by Anhui global pharmaceutical (HQ China), Inc. (http://www.ccpie.org/news/download/pharm-news-6.pdf)
Tebipenem	2009	1	originated by Wyeth KK (HQ Japan, formally known as Lederle Japan), marketed in Japan by Meiji Seika Pharma Co-Ltd (HQ Japan) after acquiring rights from Wyeth KK (http://www.meiji.com/global/investors/results-and-presentations/annual-reports/pdf/2004/annual-reports_2004_ms_en.pdf). Takeda and US Group Cyanamid created Lederle Japan, US Group Cyanamid was bought by Wyeth in 1994 leading to Lederle Japan changing name Wyeth Lederle Japan in 1998 and to Wyeth KK in 2003. Wyeth bought Takedas rights to Wyeth KK in 2007.
Telavancin	2009	1	originated by Theravance Inc (HQ US) who entered a collaboration with Astellas Pharma Inc. (HQ Japan) to develop and commercialize Telavancin worldwide (https://www.astellas.us/docs/us/VIBATIV%20cSSSI%20Launch%20Press%20Release%202009Nov5%20Final.pdf?v=1). Theravence is listed as the marketing company in the US. Theravance Biopharma Ireland Limited (subsidiary to Theravance) holds marketing rights in the EU (http://www.ema.europa.eu/ema/index.jsp?curl=pages/medicines/human/medicines/001240/human_med_001467.jsp&mid=WC0b01ac058001d124, https://www.bloomberg.com/research/stocks/private/snapshot.asp?privcapId=305360349). SciClone Pharmaceuticals (HQ US, Chinese focus) holds rights to China, Hong Kong, Macau, Taiwan, Vietnam (http://investor.sciclone.com/releasedetail.cfm?releaseid=914990, https://www.sec.gov/Archives/edgar/data/880771/000088077116000135/scln-20151231x10k.htm). R-Pharm (HQ Russia) holds rights to Russia, Ukraine, other member countries of the Commonwealth of Independent States, and Georgia (http://investor.theravance.com/static-files/9482d663-2071-4c40-9d0a-36c505b255ca). Pendopharm (HQ Canada) holds right in Canada (http://pendopharm.com/newsroom/pendopharm-launches-vibativ-in-canada/)
Ceftolozane/ Tazobactam	2014	1	originated by Astellas (HQ Japan), globally marketed by Cubist who obtained rights (to all but the Asia-Pacific region) when they aqcuired Calixa Therapeutics (HQ US) (which had bought rights from the developer Astellas)(http://www.frazierhealthcare.com/portfolio/calixa-therapeutics), and by buying Asia-Pacific marketing rights directly from Astellas (http://www.businesswire.com/news/home/20130311005458/en/Cubist-Obtains-Remaining-Rights-Ceftolozane-Astellas). Cubist was later bought by Merck (US) who marketed the drug in the EU (http://www.ema.europa.eu/ema/index.jsp?curl=pages/medicines/human/medicines/003772/human_med_001917.jsp&mid=WC0b01ac058001d124)
Dalbavacin	2014	1	originated by Vicuron Pharmaceuticals, Inc. (HQ US) (https://www.bloomberg.com/research/stocks/private/snapshot.asp?privcapId=36229). Pfizer (HQ US) bought rights to dalbavancin from Vicuron (http://www.outsourcing-pharma.com/Preclinical-Research/Pfizer-Vicuron-in-1.9-billion-merger), rights was then passed on to Durata Therapeutics (HQ US) (a spin off from Pfizer) which marketed dalbavancin in the US (http://www.news-medical.net/news/20091221/Durata-Therapeutics-acquires-Vicuron-Pharmaceuticals.aspx, https://www.accessdata.fda.gov/drugsatfda_docs/appletter/2014/021883Orig1s000ltr.pdf). Actavis (HQ US) later bought Durata (https://www.sec.gov/Archives/edgar/data/1544116/000119312514364695/d800599dex992.htm) as well as Allergan (HQ Ireland) (https://www.allergan.com/news/news/thomson-reuters/actavis-completes-allergan-acquisition), changing the name to Allergan plc. which currently stands as holding the marekt authorization in the US. Allergan Pharmaceuticals International Ltd (Ireland) holds the market authorization in Europe (http://www.ema.europa.eu/ema/index.jsp?curl=pages/medicines/human/medicines/002840/human_med_001848.jsp&mid=WC0b01ac058001d124). Allergan licensed rights to Cardiome Group (HQ Canada) to commercialize in Canada, France, the UK, Germany, Belgium, Nordic nations, certain other European nations, various Middle Eastern nations (http://www.prnewswire.com/news-releases/cardiome-and-allergan-announce-xydalba-dalbavancin-licensing-agreement-in-international-markets-578314031.html), while Angelini (HQ Italy) holds rights to Italy, Spain, and eastern European countries (https://www.thepharmaletter.com/article/angelini-inks-deal-with-allergan-on-dalbavancin)
Oritavancin	2014	1	originated by Eli Lilly (HQ US) originally licensed to Intermune (HQ US), which sold oritavancin to Targanta Therapeutics (HQ US) who was denied EMA authorization. Targanta, including oritavancin, was then bought by The Medicines Company (http://www.themedicinescompany.com/investors/sec-filing/9437377/aHR0cDovL2FwaS50ZW5rd2l6YXJkLmNvbS9maWxpbmcueG1sP2lwYWdlPTk0MzczNzcmRFNFUT0xJlNFUT0xMyZTUURFU0M9U0VDVElPTl9QQUdFJmV4cD0mc3Vic2lkPTU3, https://www.forbes.com/2009/01/13/targanta-medicines-antiobiotics-markets-equity-cx_lal_0113markets26.html). Oritavancin is currently marketed by The Medicines Company UK Ltd in Europe and the US
Garenoxacin	2007	2	marketed by the originator Astellas, Taisho Toyama and Toyama Chemical (HQs Japan) which holds rights to China, Japan, South Korea (https://www.toyama-chemical.co.jp/eng/news/news0331e.html). Global rights (a part from previously listed counties) were sublicensed to Schering-Plough (HQ US) who failed to get EMA market authorization

			(http://www.ema.europa.eu/ema/index.jsp?curl=pages/news_and_events/news/2009/11/news_detail_000106.jsp&mid=WC0b01ac058004d5c1). Schering-Plough was bought by Merck (HQ US) in 2009
Sitafloxacin	2008	2	originated and marketed by Daiichi Sankyo Company (HQ Japan) (http://www.daiichisankyo.com/media_investors/media_relations/press_releases/detail/005667.html)
Tedizolid	2014	2	originated by Dong-A Pharmaceutical (HQ South Korea) holds marketing rights in South Korea (http://en.donga-st.com/B02.da?method=prdtMedicineList). Global rights were sublicensed to Trius Therapeutics (HQ US) which sublicensed rights to Asia (excl. South Korea), Africa, South America to Bayer (HQ Germany) (https://www.sec.gov/Archives/edgar/data/1356857/000119312513105369/d444102d10k.htm). Cubist (HQ US) bought Trius incl. its rights to tedizolid and marketed tedizolid in the US (http://files.shareholder.com/downloads/ABEA-43S70D/2240349150x0x680687/c4f945be-0429-4f54-82df-6488936c653b/Trius_Press_Release_7-30-13.pdf). Merck (HQ US) bought Cubist and holds marketed in Europe (http://www.ema.europa.eu/ema/index.jsp?curl=pages/medicines/human/medicines/002846/human_med_001856.jsp&mid=WC0b01ac058001d124)
Balofloxacin	2002	3	originated by Chugai Pharmaceutical and CIBA (https://www.ncbi.nlm.nih.gov/pubmed/12669387), marketed by Choongwae Pharma, now JW Pharmaceuticals (HQ South Korea) (http://www.cwp.co.kr/pharma/en/intro/history.jsp, https://www.nextpharmajob.com/resourcearticles.aspx?rid=62&)
Pazufloxacin	2002	3	originated by Toyama Chemical (http://www.mt-pharma.co.jp/e/release/nr/2010/pdf/eMTPC_P100723.pdf), marketed by Mitsubishi Tanabe Pharma Corporation (formally known as Welfide Corp) (https://www.ncbi.nlm.nih.gov/pubmed/11249595)
Biapenem	2002	3	originated by Wyeth KK (HQ USA), marketed by Meiji Seika Pharma Co.Ltd (HQ Japan) (http://www.meiji.com/global/about-us/corporate-profile/meiji-holdings/pdf/HD_all.pdf, http://www.tadinter.com/en/achive.html)
Ceftobiprole	2008	5	originated by Hoffmann – La Roche (HQ Switzerland), marketed by Basilea Pharmaceuticals (HQ Switzerland) (a spin-off from Hoffmann-La Roche) (http://www.basilea.com/News-and-Media/Basilea-announces-launch-of-antibiotic-Zevtera-ceftobiprole-medocaril-in-Germany/59334b49-49f7-9596-c42f-93124a0d5979/, http://www.basilea.com/Portfolio/Ceftobiprole/). Basilea granted Janssen Cilag GmbH International (HQ Switzerland) (subsidiary of Johnson and Johnson, HQ US, since 1990) global rights in 2005 (http://www.basilea.com/News-and-Media/European-CHMP-adopts-negative-opinion-on-ceftobiprole/340/). Janssen Cilag was denied EMA authorization in 2010 and rights returned to Basilea (http://www.basilea.com/News-and-Media/Discontinuation-of-sale-of-ceftobiprole-in-Switzerland/373/)
Prulifloxacin	2002	14	originated by Nippon Shinyaku (HQ Japan) who sublicensed to a number of companies (http://www.nippon-shinyaku.co.jp/official/ir/library/annualreport2003.pdf, https://www.nippon-shinyaku.co.jp/english/company_profile/news.php?id=2336). Meiji Seika Pharma Co.,Ltd (HQ Japan) holds rights in Japan (http://www.meiji-seika-pharma.co.jp/english/corporate/history/), Yuhan Corporation (HQ South Korea) holds rights in South Korea, Angelini Group (HQ Italy) holds rights in Europe (http://www.nippon-shinyaku.co.jp/official/english/news/ir_meeting_131107.pdf), Optimer Pharmaceuticals, Inc. (HQ US) holds rights in the US (http://www.businesswire.com/news/home/20090224005441/en/Optimer-Pharmaceuticals-Announces-Positive-Results-Phase-3)
Dalfopristin/ Quinupristin	1999	21	originated by Rhone-Poulenc Rorer (ref. http://en.sanofi.com/Images/16120_20F_Doc-Ref_2001_Aventis_EN.pdf), marketed by Aventis (formed in the merger between Hoechst AG and Rhone-Poulenc Rorer) who got it approved in the US but later sublicensed rights to the US and other specific markets to Monarch Pharmaceuticals Inc., a subsidiary of King Pharmaceuticals. King holds marketing authorization in the US (http://www.evaluategroup.com/Universal/View.aspx?type=Story&id=35311). (King Pharmaceuticals is now owned by Pfizer: https://www.sec.gov/Archives/edgar/data/912183/000104746914001280/a2218356z10-k.htm). European rights were sublicensed to Nordic Pharma in 2004 (http://www.evaluategroup.com/Universal/View.aspx?type=Story&id=111492)
Gemifloxacin	2003	28	originated by LG Life Sciences (a spin-off from LG CHEM LTD), marketed in the US by LG Chem Ltd (HQ South Korea). LG Chem Ltd sublicensed rights to Oscient pharmaceuticals (HQ US) (http://www.evaluategroup.com/Universal/View.aspx?type=Story&id=131864) who sublicensed European rights to Menarini (HQ Italy) (https://pipelinereview.com/index.php/200701059035/Small-Molecules/Oscient-Pharmaceuticals-and-Menarini-Group-Enter-into-European-Commercialization-Agreement-for-FACTIVE-Tablets.html). Menarini did however not recieve marketing authorization in Europe. Oscien Pharmaceuticals sublicensed remaining areas to a number of companies: Abott Canada (HQ Canada) holds the rights to the Canadian market (http://www.evaluategroup.com/Universal/View.aspx?type=Story&id=118777), Pfizer (HQ US) holds the right in Mexico (http://www.evaluategroup.com/Universal/View.aspx?type=Story&id=97990)
Gatifloxacin	1999	30	originated by Kyorin Pharmaceuticals (http://www.kyorin-pharm.co.jp/en/company/history.shtml, https://www.bms.com/trademarks.html), marketed by Bristol-Myers Squibb
Ceftaroline	2010	31	originated by Takeda Chemical Industries, Ltd. (HQ Japan) sublicensed the marketing rights in Japan to Dainippon Sumitomo Pharma Co., Ltd. (HQ Japan) (http://www.ds-pharma.com/ir/news/pdf/ene20110330_2.pdf) , while the global rights was sublicensing to Peninsula Pharmaceuticals (HQ US) which sold rights to Cerexa (HQ US), Inc. Forest Laboratories (HQ US) took over right when they bought Cerexa in 2007 and marketed in the US (http://www.annualreports.com/HostedData/AnnualReportArchive/f/NYSE_FRX_2011.pdf,

			http://www.fiercebiotech.com/biotech/forest-buys-cerexa-480m-deal). In 2009 Forest entered an agreement with AstraZeneca (HQ UK) to co-develop and commercialize in all markets outside the U.S., Canada and Japan (https://www.allergan.com/news/news/thomson-reuters/allergan-receives-fda-approval-of-teflaro-ceftaro, https://www.sec.gov/Archives/edgar/data/38074/000003807406000052/exhibit99.htm. AstraZeneca sold rights to markets outside of US to Pfizer (https://www.astrazeneca.com/media-centre/press-releases/2016/AstraZeneca-to-sell-small-molecule-antibiotics-business-to-Pfizer-24082016.html)
Telithromycin	2001	43	originated and marketed by Aventis (HQ France) (currently Sanofi after the merger between Sanofi and Aventis (http://en.sanofi.com/Images/20524_Sanofi_RA_2008_A_EN.pdf, http://www.ema.europa.eu/ema/index.jsp?curl=pages/medicines/human/medicines/000354/human_med_000873.jsp&mid=WC0b01ac058001d124 , http://www.sanofi.us/l/us/en/layout.jsp?cnt=CC3802F6-EABB-4B10-A43D-69E85CEF340B)
Doripenem	2005	44	originated by Shionogi (HQ Japan) which holds the rights to the Japanese market (http://www.shionogi.eu/media/37330/29112011.pdf). Shionogi sublicensed global rights to Peninsula Pharmaceuticals, Inc. (HQ US). Johnson & Johnson Pharmaceutical obtained global rights by acquiring Peninsula Pharmaceuticals (http://www.investor.jnj.com/releasedetail.cfm?releaseid=160602). Johnson and Johnsons subsidiary Janssen-Cilag International NV marketed doripenem in Europe but later withdrew the antibiotic. Due to difficulties Johnson & Johnson gave global marketing rights back to Shinogi in 2013 (http://www.csringreece.gr/files/reports/en/2013/2013-JNJ-Citizenship-Sustainability-Report-FINAL061914.pdf, http://files.shareholder.com/downloads/JNJ/3012794104x0x733042/ddd2abd5-2cc6-41d2-8acb-ec2a967727e4/ar2013_JNJ.pdf)
Daptomycin	2003	47	originated by Eli Lilly (HQ US), marketed by Cubist (HQ US) (https://www.ncbi.nlm.nih.gov/pmc/articles/PMC1661656/). Cubist sublicensed the European rights to Chiron Corporation Ltd. (http://www.businesswire.com/news/home/20031003005112/en/Cubist-Pharmaceuticals-Chiron-Corporation-Announce-CUBICIN-International) which was acquired by Novartis (HQ Switzerland) (https://www.sec.gov/Archives/edgar/data/912183/000104746912001597/R9.htm). Merck sublicensed rights to Japan. The rights to other parts of Asia incl. China and selected Middle East countries to AstraZeneca (HQ UK) (https://www.astrazeneca.com/content/dam/az/Investor_Relations/annual-reports-homepage/2008-Annual-Report-English.pdf), right to the Canadian market to Sunovion Pharmaceuticals Canada (HQ Canada), and rights to Japan to Banyu Pharmaceutical Co., Ltd. Both Cubist and Banyu is now owned by Merck (https://www.sec.gov/Archives/edgar/data/912183/000104746914001280/R10.htm). Merck is currently listed as the holder of marketing authorization in Europe
Ertapenem	2002	65	originated by AstraZeneca (HQ UK), marketed by Merck Sharp & Dohme Ltd (https://www.astrazeneca.com/content/dam/az/PDF/Q1_2016_results_press_release.pdf, https://www.accessdata.fda.gov/drugsatfda_docs/nda/2001/21337_Invanz_admindocs.pdf)
Tigecycline	2005	65	originated by Wyeth (HQ US) who marketed Tygacil in the US. Pfizer later obtained rights by acquiring Wyeth (http://press.pfizer.com/press-release/pfizer-acquire-wyeth-creating-worlds-premier-biopharmaceutical-company, https://www.clinicaltrials.gov/ct2/show/NCT00079989, http://www.pfizer.com/products/product-detail/tygacil)
Linezolid	2000	70	originated and marketed by Pharmacia & Upjohn (https://www.ncbi.nlm.nih.gov/pubmed/11249571). The company was later bought by Pfizer (http://www.pfizer.com/about/history/pfizer_pharmacia, http://www.pfizer.com/products/product-detail/zyvox)
Moxifloxacin	1999	74	originated and marketed by Bayer (HQ Germany). Shinogi obtained marketing rights in Japan (http://www.investor.bayer.de/en/nc/news/archive/investor-news-2005/investor-news-2005/bayer-wins-approval-for-aveloxR-moxifloxacin-in-japan/, http://www.shionogi.co.jp/ir/pdf/all04.pdf), but transferred rights back to Bayer in 2010 (http://www.shionogi.co.jp/en/company/news/2010/pmrltj00000014ed-att/e_100510_1.pdf)

Companies in charge of development and marketing of the NCEs. MA= market authorization. In addition to the references listed in the table, information was gathered from the AdisInsight database and the Annual Reports in Medicinal Chemistry volume 36-46. Note. This table does not attempt to give a full list of all companies involved in each NCE, but rather provide an insight in the complexity of market introduction.
