# Supplementary material for: Introduction and geographic availability of new antibiotics approved between 1999 and 2014
Source: PLoS One. 2018 Oct 16;13(10):e0205166. doi: 10.1371/journal.pone.0205166 (PMC6191083; doi:10.1371/journal.pone.0205166)
Supplement: S3 Table — (PDF) [file pone.0205166.s003.pdf]

S3 Table. EMA and FDA market authorization dates

NCE	EMA market authorization: centralized authorization procedure	FDA market authorization
antofloxacin	no application	-
balofloxacin	no application	-
Biapenem	no application	-
ceftaroline	23.08.2012	29.10.2010
ceftobiprole	Refused (16.09.2010)	-
ceftolozane	18.09.2015	19.12.2014
dalbavancin	19.02.2015	23.05.2014
dalfopristin/ quinupristin	no application	21.09.1999
daptomycin	19.01.2006	12.09.2003
doripenem	25.07.2008	12.10.2007
ertapenem	18.04.2002	21.11.2001
garenoxacin	Refused (25.07.2007)	-
gatifloxacin	no application	Withdrawn (2006)
gemifloxacin	Refused (17.06.2009)	04.04.2003
linezolid	no application	18.04.2000
moxifloxacin	no application	10.12.1999
oritavancin	19.03.2015	06.08.2014
pazufloxacin	no application	-
prulifloxacin	no application	-
sitafloxacin	no application	-
tebipenem	no application	-
tedizolid	23.03.2015	20.06.2014
telavancin	02.09.2011	11.09.2009
telithromycin	09.07.2001	01.04.2004
tigecycline	24.04.2006	15.06.2005

EMA and FDA market authorization dates. FDA does not report on NCEs not authorized. European countries have registered sales of ceftobiprole, dalofopristin/quinupristin, gatifloxacin, gemifloxacin, linezolid, moxifloxacin, and prulifloxacin, indicating that these NCEs have been approved through the mutual recognition procedure. Doripenem and telithromycin were later withdrawn from market by the pharmaceutical companies holding the market authorization.
