# Supplementary material for: Introduction and geographic availability of new antibiotics approved between 1999 and 2014
Source: PLoS One. 2018 Oct 16;13(10):e0205166. doi: 10.1371/journal.pone.0205166 (PMC6191083; doi:10.1371/journal.pone.0205166)
Supplement: S4 Table — (PDF) [file pone.0205166.s004.pdf]

S4 Table. Company size measured in net sales and number of employees

NCE	Product name	MA year	Originator	USD million	Employees	Developed and marketed by	USD millions	Employees	**Total number of countries entered**
moxifloxacin	Avelox®	1999	Bayer	7444.97	120400	Bayer	7639.93	120400	**75**
linezolid	Zyvox®	2000	Pharmacia	9314.00	44300	Pharmacia	13837.00	no data	**70**
ertapenem	Invanz®	2002	Astra Zeneca	17841.00	15000	Merck	19946.20	77300	**65**
tigecycline	Tygacil®	2005	Wyeth	17875.00	49732	Wyeth	17875.00	49732 (2005)	**65**
daptomycin	Cubicin®	2003	Eli Lilly	11855.90	46100	Cubist	3.716	290	**47**
doripenem	Finibax®	2005	Shionogi	1531.35	4293	Shinogi/ Peninsula (owned by J&J)	22322.00 (J&J)	115600 (J&J)	**44**
telithromycin	Ketek®	2001	Aventis	15726.35	no data	Aventis	16138.19	no data	**43**
ceftaroline	Teflaro®	2010	Takeda	17212.25	19654	Forest Laboratories Inc.	3903.52	5000	**31**
gatifloxacin	Tequin®	1999	Kyorin Pharmaceuticals	379.43	1000	Bristol-Myers Squibb	16000.00	54500	**30**
gemifloxacin	Factive®	2003	LG Life Sciences	6813.65	no data	Oscient	6.612	94	**28**
dalfopristin/ quinupristin	Synercid ®	1999	Rhone-Poulenc-Rorer	4677.78	65180	Aventis	4456.86	65180	**21**
prulifloxacin	Sword®	2002	Nippon Shinyaku	342.15	1800	Nippon Shinyaku and Meiji Seika	903.96	4418 (Meiji)	**14**
ceftobiprole	Zeftera®	2008	Roche	18309.66	54141	Basilea/ J&J	9.91 (Basilea) 24567.00 (pharma J&J)	300 (Basilea) 118700 (J&J)	**5**
balofloxacin	Q-roxin®	2002	Chugai Pharmaceutical	1900.73	6282	Choongwae (now JW Pharmaceuticals)	345.07	no data	**3**
biapenem	Omegacin ®	2002	Wyeth KK (Wyeth)	14584.04	52762	Meiji Seika	903.96	4418	**3**
pazufloxacin	Pasil®	2002	Toyama Chemical	0.2315	1662	Toyama Chemical/Mitsubishi	1869.98	5339 (Mitsubishi)	**3**
garenoxacin	Geninax®	2007	Toyama Chemical	0.15	1025	Toyama /Taisho/Astellas	836.23 (Taisho) 8314.86 (Astellas)	5327 (Taisho) 13 900 (Astellas)	**2**
sitafloxacin	Gracevit®	2008	Daiichi Sankyo	8929.42	28895	DaiichiSankyo	7972.26	28895	**2**
tedizolid	Sivextro®	2014	Dong-A Pharmaceutical	401.64	2563	Cubist	1054.44	873	**2**

antofloxacin	优朋®	2010	Shanghai Institute Of Materia Medica	academia	academia	Anhui global pharmaceutical	academia	academia	**1**
ceftolozane/ tazobactam	Zerbaxa®	2014	Astellas	9836.04	17649	Cubist	1054.44	873	**1**
dalbavancin	Dalvance ®	2014	Vicuron Pharmaceuticals	8.372	no data	Durata (spin off from Pfizer)	0.00	68 (Durata)	**1**
oritavancin	Nuvocid®	2014	Eli Lilly	17269.00	39135	The Medicines Company	724.40	727	**1**
tebipenem	Orapenem ®	2009	Wyeth K.K (Wyeth)	19025.4	no data	Meiji Seika Pharma	1468.38	14168	**1**
telavancin	Vibativ®	2009	Theravance	24.37	194	Theravance/Astellas	11110.55	15161 (Astellas)	**1**

Net sales in USD (in millions) and number of employees the year of market entry, of the companies in charge of development and marketing of the NCEs.
